# ﻿Rediscovery of *Primulabrachystoma* (Primulaceae), a rare species endemic to Gaoligong Mountain of Chinese-Burma frontier

**DOI:** 10.3897/phytokeys.227.103746

**Published:** 2023-05-29

**Authors:** Xiao-Qi Jiang, Na Zhang, Zhi-Kun Wu

**Affiliations:** 1 Department of pharmacy, Guizhou University of Traditional Chinese Medicine, Guiyang, 550025, Guizhou, China Guizhou University of Traditional Chinese Medicine Guiyang China

**Keywords:** Endangered species, Gaoligong Mountain, *Primula*, Primulaceae, rediscovery

## Abstract

The rare *Primulabrachystoma* W.W.Sm. is an endemic species confined to Gaoligong mountain of Chinese-Burma frontier, which has been rediscovered from the same region after nearly 100 years. In total, 11 specimens from Gaoligong Mountain have been found in the herbaria worldwide, since its first collection in 1920 by Farrer, Reginald John. Previously, this species was described as homostylous but our finding shows the species also exhibited heterostyly. A complete description of the species, the distribution, morphological comparison and identification key from closely related species are provided here. An assessment of its conservation status suggests that the species is ‘Endangered’ (EN).

## ﻿Introduction

*Primulabrachystoma* W.W.Sm. was discovered by Farrer, Reginald John from Shing Hong of Burma in Gaoligong Mountain of Chinese-Burma frontier under the collection number Farrer 1635 in 1920, then was described as a new species in 1923 by Smith (Smith and Forrest 1923). A few of these type specimens were preserved as *P.brachystoma* in the Royal Botanic Gardens, Kew (K) and the Natural History Museum, London (BM). Others were preserved in the herbarium of Edinburgh. This species was considered closely related to Primulaprenanthasubsp.prenantha Balf.f. & W.W.Sm. and Primulaprenanthasubsp.morsheadiana (Kingdon-Ward) F.H.Chen & C.M.Hu, but the acute leaf apex makes it easily distinguishable from these two other species (Smith and Fletcher 1941). In the description of P.prenanthasubsp.prenantha in the Flora Reipublicae Popularis Sinicae (Hu 1990), it was claimed that its closely related species *P.brachystoma* did not distribute to China. However, when we reviewed the specimens from key Herbaria (BM, E, IBSC, K, KUN, P, PE), we found one plant collected at Tsuga on the way from Gongshan downtown to Dulong River, Yunnan, east slope of Gaoligong mountain, which was identified as *Primulabrachystoma* W.W.Sm. by Professor Chi-Ming Hu (Qinghai-Tibet team 8648, PE), and another specimen with a similar number collection was not identified (Qinghai-Tibet team 8648, PE). In contrast, two specimens with the same collection number at KUN were identified as PrimulaelegansForrestvar.maculosa H.Chuang. However, a comparison with the type specimen of *P.brachystoma* indicates that all these specimens belong to this species; therefore, our observation suggests that *P.brachystoma* is also distributed in China.

**Figure 1.**
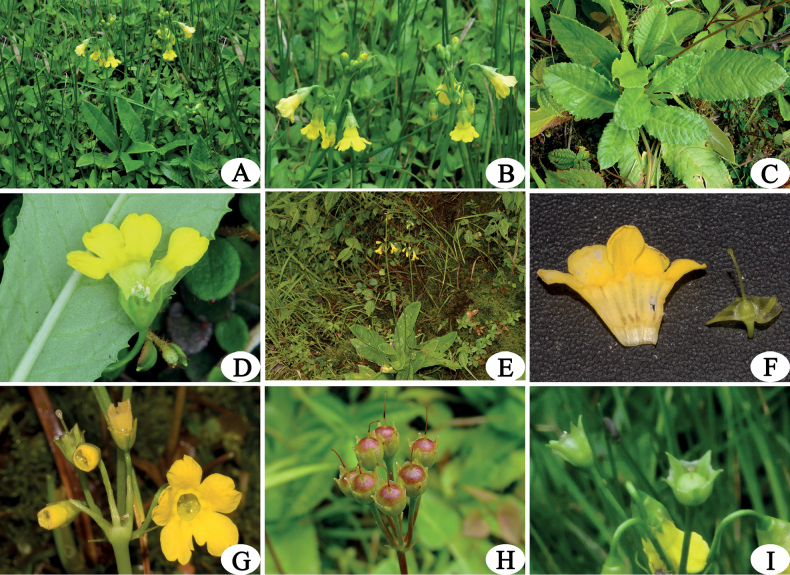
*Primulabrachystoma***A**–**D** homostylous flower individuals: **A** habit **B** inflorescence **C** leaf blade **D** homostylous flower **E**–**I** heterostylous flower individuals: **E** habit **F**–**G** long style of heterostylous flower (pin) **H** fruits of pin flowers **I** fruits of thrum flowers. Photographed by Z. K. Wu.

*Primulabrachystoma* was originally described as homostylous by Smith (Smith and Forrest 1923). During a botanical expedition in the regions of Gaoligong Mountain in May 2015, we found a homostylous *Primula* with an acute leaf apex and regularly denticulate at margin, campanulate calyx and yellow corolla with annulus marked, on the western slopes of the Gaoligong Mountains near Dulong River. After a comparison with type specimens, the plant matched *P.brachystoma* (Fig. 1A–D). Another heterostylous *Primula* with an acute leaf apex and regularly denticulate at margin, campanulate calyx and yellow corolla with annulus marked, was found in Shibali and Yaping of Fugong county during a botanical expedition in Gaoligong Mountain of Fugong Xian, Yunnan, China in 2018. After comparing with the type specimens, excluding the heterostylous flowers, all its other characteristics match the description of *P.brachystoma*. Considering that some species in the genus *Primula* have both homostylous and heterostylous flowers such as *Primulachungensis* Balf.f. & Kingdon-Ward, *Primulaoreodoxa* Franch., *Primulapolonensis* Kingdon-Ward and *Primulasinensis* Sabine ex Lindl. etc (Hu 1990; Bawri et al. 2015), the plant from Shibali and Yaping could be *P.brachystoma* (Fig. 1E–I). Therefore, we believe that *P.brachystoma*, having both homostylous and heterostylous flowers, is similar to its closely allied species *P.polonensis* and *P.chungensis* in the same section. This paper provides a complete morphological description, distribution, morphological comparison and identification key from closely related species. The threat status of *P.brachystoma* through field surveys and review of type specimens, as well as color photographs, are also provided to facilitate proper identification of the species (Figs 1, 3).

**Figure 2.**
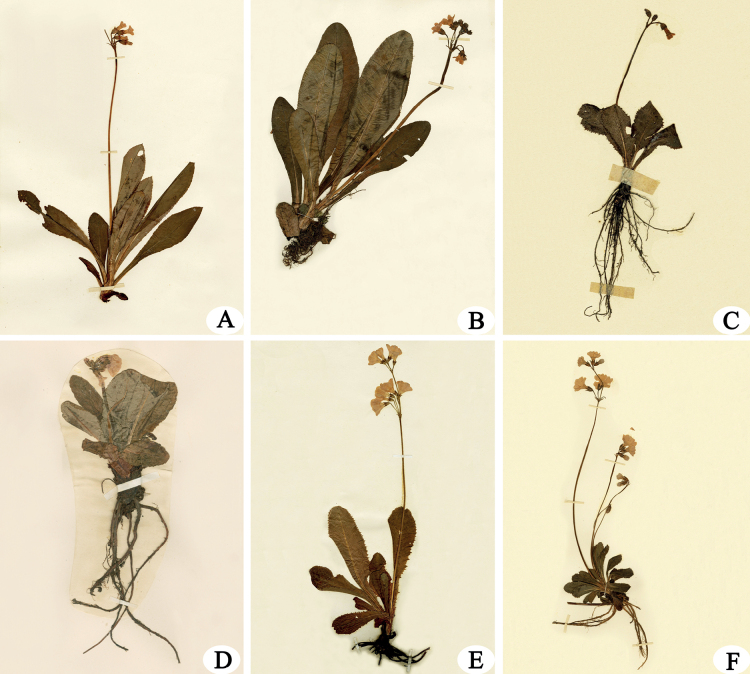
Specimens of *P.brachystoma* and its related species **A** holotype of *P.brachystoma* (Farrer 1635, E, https://data.rbge.org.uk/herb/E00024386) **B** isotype of *P.polonensis* (Kingdon-Ward 8388, E, https://data.rbge.org.uk/herb/E00024403) **C** isosyntype of P.prenanthasubsp.prenantha (G. King’s, E, https://data.rbge.org.uk/herb/E00259794) **D** type of P.prenanthasubsp.morsheadiana (Ward, F. K. 5858, K, http://apps.kew.org/herb/K000750095) **E** holotype of *P.serratifolia* (Forrest 1816, E, https://data.rbge.org.uk/herb/E00024076) **F** syntype of *P.melanodonta* (Kingdon-Ward 7042, E, https://data.rbge.org.uk/herb/E00531116).

**Figure 3.**
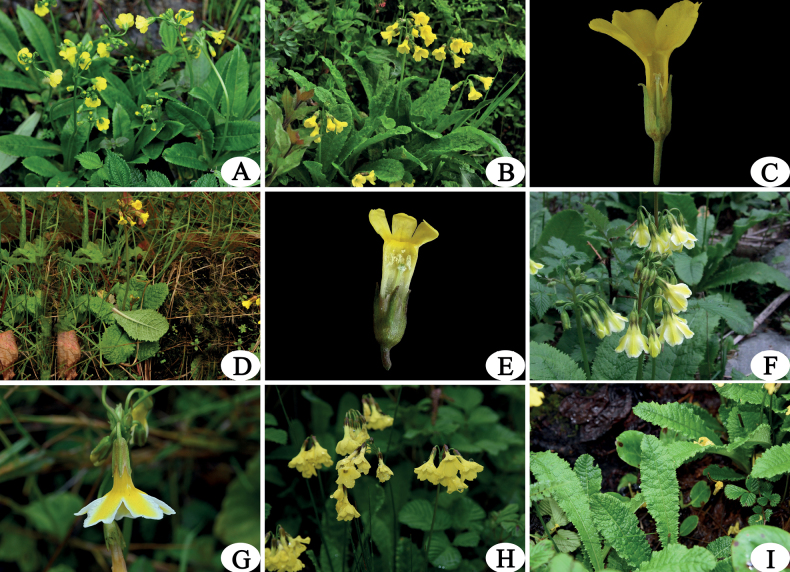
*Primulabrachystoma* and four of its close taxa **A***P.brachystoma***B**P.prenanthasubsp.morsheadiana (from its type locality: pass of Duoxiongla, Xizhang) **C** flower of P.prenanthasubsp.morsheadiana (homostyly) **D**P.prenanthasubsp.prenantha (from Gaoligong Mountain, Yunnan) **E** flower of P.prenanthasubsp.prenantha (homostyly) **F***P.serratifolia* (from its type locality: Cangshan Mountain, Yunnan) **G** flower of *P.serratifolia* (flower are biocolorous) **H***P.melanodonta* (from Gaoligong Mountain, Yunnan, flowers are concolorous) **I** leaf blade of *P.melanodonta*. Photographed by Z. K. Wu.

## ﻿Materials and methods

We collected fresh material and specimens of *P.brachystoma* from Gongshan county, Yunnan on the way to the Dulong River in May 2015 while travelling from Yaping and Shibali of Fugong county in July 2018. The identity of our plant collection has been confirmed by consulting the original description and online images of the type specimens from key Herbaria (BM, E, K). Complete morphological characters of the species were measured using a vernier calliper. The voucher specimens are stored at KUN. For comparison purposes, specimens of closely related species, *P.polonensis*, P.prenanthasubsp.prenantha, P.prenanthasubsp.morsheadiana, *P.serratifolia* Franch., *Primulamelanodonta* W.W.Sm. from the key herbaria of China (IBSC, KUN, PE), type specimens’ images online of the closely related species from BM, E, K, P, and relevant literature (Smith et al. 1977; Hu 1990; Hu and Kelso 1996) were also consulted. The conservation status of *P.brachystoma* was assessed using the guidelines for IUCN Red List categories and criteria (IUCN 2022; IUCN Standards and Petitions Committee 2022).

## ﻿Taxonomic treatment

### Primula brachystoma

Taxon classificationPlantaeEricalesPrimulaceae

W. W. Sm.

E0DB06CB-9D96-52B4-9260-16E91B107CB3

Primula brachystoma W. W. Sm. in Notes Roy. Bot. Gard. Edin., xiv, 35 (1923); W. W. Sm. et Forrest, ibid., xvi, 17 (1928), and in Journ. Roy. Hort. Soc. London, liv, 43 (1929); W. W. Smith et Fletcher in Trans. Bot. Soc. Edinb. xxxiii: 166 (1941). Type: Burma, Shing Hong region, 20 June 1920, R. J. Farrer 1635 (holotype E! E00024386; isotypes BM, BM000996925, K, K000732874).

#### Description.

A perennial herb, completely glabrous and efarinose, with numerous robust roots. ***Leaves*** forming a rosette; leaf blade oblanceolate to sublanceolate, 6–12 cm long, 2–4 cm broad, acute and shortly apiculate at the apex, petiole very short or as long as 1/3 of the blade, base attenuate with broadly winged petiole, margin regularly fine dentate, teeth triangular, apex acute and subulate, leaf abaxially with prominent midrib and conspicuous lateral veins, and inconspicuous mesh vein. ***Scapes*** slightly slender, 15–25 cm long; umbels 1 (or rarely 2), 2–7 flowered. ***Bracts*** linear-lanceolate, 0.6–0.8 cm long. ***Pedicel*** slightly recurved when flowering, erect when fruiting, 0.8–1 cm long in flowering, up to 1.5 cm long in fruiting. ***Calyx*** campanulate, green, 5–7 mm long, with 5 ribs, splitting slightly less than 1/3 of its full length, lobes triangular, apiculate at the apex. ***Flowers*** homostylous or heterostylous in different populations, corolla funnel-shaped, yellow, tube 8–10 mm long, with a marked annulus, limb 1–1.5 cm wide, lobes sub-quadrangular to obovate, 4–5 mm long, shallowly notched; homostylous flowers: the stamens are inserted in the middle of the corolla tube, filament ca. 0.5 mm long, anther ca. 0.8–1 mm long, yellowish white, the style reaches to the level of the stamens; heterostylous flowers: in long-styled flowers the style nearly reaches the annulus and the stamens are inserted towards the base of the corolla, in short-styled flowers the stamens are inserted slightly below the annulus and the style is shorter than the calyx, the filament and anther as those in homostylous flowers. ***Capsule*** globose, green in young fruiting time and pale purple in mature fruiting time, 5–6 mm long, as long as or slightly shorter than calyx, 4–5 mm in diameter.

#### Distribution and habitat.

It is found on Gaoligong Mountain on the border between China and Burma. One homostylous population is at the type locality Shing Hong of Burma and two homostylous populations are at Sandui and Tsuga of Gongshan county, China; the other two heterostylous population are at Sibali and Luodigolu, Yaping of Fugong county, China. It grows along moist streams or on wet grassy slopes at forest margins, 2500–3000 m above sea level.

#### Additional specimens examined.

China, Gongshan Xian, Dulongjiang Xiang: Sandui [27°42′56.112′′N, 98°25′24.048′′E, 2580 m], May 2015, ZKWU 2015036 (KUN!); China, Fugong Xian, Lishadi Xiang: Yaduo Cun [27°10′36′′N, 98°44′55.9′′E, 2830 m], 6 August 2005, Gaoligong Shan Biodiversity Survey 26554 (KUN!); China, Fugong Xian, Lumadeng Xiang: Yaping Cun [27°10′3′′N, 98°46′17.7′′E, 2510 m], 16 August 2005, Gaoligong Shan Biodiversity Survey 28479 (KUN!); China, Fugong Xian, Lishadi Xiang: Yaduo Cun [27°10′1.8′′N, 98°46′24.8′′E, 2520 m], 16 August 2005, Gaoligong Shan Biodiversity Survey 28435 (KUN!); China, Gongshan Xian: Tsuga on the way from Gongshan Xian to Dulong River, east slope of Gaoligong mountain, 26 July 1982, Qinghai-Tibet team 8648 (PE, KUN!).

#### Provisional conservation status.

*Primulabrachystoma* is neither listed in the IUCN Red List (IUCN 2022), nor in the threatened Species List of China’s Higher Plants (Qin et al. 2017). The authors have conducted field surveys in the regions of Gaoligong mountains many times and discovered only three populations of *Primulabrachystoma* in Fugong county and Gongshan county. Surveys from other plant hunters also didn’t find more populations in this area. We estimated the extent of occurrence of the species to be less than 1000 km^2^, and the adult individuals as fewer than 1000. The sites where the known populations grow are also places for grazing, so they face a strong threat from human activities. Accordingly, we evaluate the species as Endangered (EN B1ab(iii)), considering the IUCN standards (IUCN Standards and Petitions Committee 2022).

## ﻿Diagnosis

Morphologically, *P.brachystoma* is similar to *P.polonensis*, P.prenanthasubsp.prenantha, P.prenanthasubsp.morsheadiana, *P.serratifolia*, *P.melanodonta* in P.sect.Proliferae Pax. Among these species, *P.brachystoma* and *P.polonensis* have both homostyly and heterostyly flowers, longer leaves with inconspicuous mesh vein on abaxial surface, and short corolla tube (8–10 mm) with a marked annulus; *P.brachystoma* differed from the latter by its leaf blade being acute and shortly apiculate, calyx splitting slightly less than 1/3 of its full length (Fig. 2A, B). *P.brachystoma* differed from the homostylous species P.prenanthasubsp.prenantha and P.prenanthasubsp.morsheadiana by the leaf blades of the latter two are both rounded at the apex, the abaxial surface of leave blade with conspicuous mesh vein, corolla tube more elongated (Fig. 2A, C, D; Fig. 3A–E). *P.brachystoma* differed from the heterostylous species *P.serratifolia* and *P.melanodonta* by the leaf blades of *P.serratifolia* and *P.melanodonta* are both rounded to obtuse at the apex, the abaxial surface of leave blade with conspicuous mesh vein (Figs 2A, E, F; 3A, F–I). The main morphological difference between *P.brachystoma* and its allies is summarized in Table 1, and the following Keys.

**Table 1.** Morphological comparisons among *P.brachystoma*, *P.polonensis*, P.prenanthasubsp.prenantha, P.prenanthasubsp.morsheadiana, *P.serratifolia* and *P.melanodonta*.

Characters	*P.brachystoma*	*P.polonensis*	P.prenanthasubsp.Prenantha	P.prenanthasubsp.Morsheadiana	*P.serratifolia*	*P.melanodonta*
**Leaf blade**	oblanceolate to sublanceolate, 6–12×2–4 cm	narrowly ovate to obovate-oblong or oblanceolate, 3–20×1.2–5 cm	oblong-obovate to obovate-elliptic, 3.5–9×1.5–3 cm	elliptic to oblanceolate leaves up to 12 cm. long and 3 cm. broad	oblong to elliptic-obovate, 6–12×1.8–5 cm	oblanceolate or obovate leaves, 3–5×1.5–2 cm
**Leaf apex**	acute and shortly apiculate	rounded to obtuse	Rounded	obtuse or rounded	rounded	rounded or obtuse
**Leaf margin**	regularly fine dentate	regularly crenulate	erose-denticulate	Irregularly denticulate	erose-denticulate	deeply dentate
**Under surface of leaf blade**	prominent midrib and conspicuous lateral veins, and inconspicuous mesh vein	prominent midrib and conspicuous lateral veins, and inconspicuous mesh vein	midrib and lateral veins prominent, and conspicuous mesh vein	midrib and lateral veins prominent, and conspicuous mesh vein	midrib and lateral veins prominent, and conspicuous mesh vein	midrib and lateral veins prominent, and conspicuous mesh vein
**Calyx**	Campanulate	tubular-campanulate	campanulate	Campanulate	tubular-campanulate	campanulate
**Calyx lobs**	splitting slightly less than 1/3 of its full length, lobes triangular	parted to middle or below, lobes narrowly oblong	parted at most to 1/3, lobes triangular	parted 1/3, into broadly triangular	Parted 1/3–1/2, 5-ribbed, lobes triangular	cut almost to the middle into ovate
**Style**	homostylous and heterostylous	homostylous and heterostylous	homostylous	Homostylous	heterostylous	heterostylous
**Capsule**	globose, as long as or slightly shorter than calyx	globose, included in calyx	subglobose, slightly longer than calyx	subglobose, as long as calyx	ovoid, nearly as long as calyx	ovoid, included in calyx

### ﻿Key for *Primulabrachystoma* and its closely related species in the P.sectionProliferae

**Table:** 

1	Flowers homostylous or heterostylous in different populations	**2**
–	Flowers only heterostylous	**5**
2	Flowers only homostylous, under surface of leaf blade with conspicuous mesh vein	**3**
–	Flowers homostylous and heterostylous in different populations, under surface of leaf blade with inconspicuous mesh vein	**4**
3	Mature corolla limb 6–9 mm wide; lobes oblong-ovate, not spreading, capsule 5 mm long, longer than the calyx	**P.prenanthasubsp.prenantha**
–	Mature corolla limb 9–12 mm wide; lobes obovate, ±spreading, capsule 7–8 mm, as long as the calyx	**P.prenanthasubsp.morsheadiana**
4	Leaf blade apex acute and shortly apiculate; bract upper part not leafy	***P.brachystoma***
–	Leaf blade apex rounded to obtuse; bract upper part leafy	***P.polonensis***
5	Leaf blade 3–5 cm, margin densely fine denticulate; pin flower style or thrum flower stamens slightly exceeding middle of corolla tube, flower concolorous	***P.melanodonta***
–	Leaf blade 6–12 cm, margin denticulate; pin flower style or thrum flower stamens exserted from corolla tube, flower bicolorous	***P.serratifolia***

## Supplementary Material

XML Treatment for Primula brachystoma

## ﻿Additional information

### Author ORCIDs

Xiao-Qi Jiang https://orcid.org/0000-0002-2101-6541

Na Zhang https://orcid.org/0000-0002-7005-2848

Zhi-Kun Wu https://orcid.org/0000-0002-4583-7782

### Data availability

All of the data that support the findings of this study are available in the main text or Supplementary Information.
